# Synthesis of ω-hydroxy dodecanoic acid based on an engineered CYP153A fusion construct

**DOI:** 10.1111/1751-7915.12073

**Published:** 2013-08-14

**Authors:** Daniel Scheps, Sumire Honda Malca, Sven M Richter, Karoline Marisch, Bettina M Nestl, Bernhard Hauer

**Affiliations:** 1 Institute of Technical Biochemistry, University of StuttgartAllmandring 31, 70569, Stuttgart, Germany; 2 Department of Biotechnology, University of Natural Resources and Life SciencesMuthgasse 18, 1190, Vienna, Austria

## Abstract

A bacterial P450 monooxygenase-based whole cell biocatalyst using Escherichia coli has been applied in the production of ω-hydroxy dodecanoic acid from dodecanoic acid (C12-FA) or the corresponding methyl ester. We have constructed and purified a chimeric protein where the fusion of the monooxygenase CYP153A from *Marinobacter aquaeloei* to the reductase domain of P450 BM3 from *Bacillus megaterium* ensures optimal protein expression and efficient electron coupling. The chimera was demonstrated to be functional and three times more efficient than other sets of redox components evaluated. The established fusion protein (CYP153A*_M. aq._-*CPR) was used for the hydroxylation of C12-FA in *in vivo* studies. These experiments yielded 1.2 g l^–1^ ω-hydroxy dodecanoic from 10 g l^–1^ C12-FA with high regioselectivity (> 95%) for the terminal position. As a second strategy, we utilized C12-FA methyl ester as substrate in a two-phase system (5:1 aqueous/organic phase) configuration to overcome low substrate solubility and product toxicity by continuous extraction. The biocatalytic system was further improved with the coexpression of an additional outer membrane transport system (AlkL) to increase the substrate transfer into the cell, resulting in the production of 4 g l^–1^ ω-hydroxy dodecanoic acid. We further summarized the most important aspects of the whole-cell process and thereupon discuss the limits of the applied oxygenation reactions referring to hydrogen peroxide, acetate and P450 concentrations that impact the efficiency of the production host negatively.

## Introduction

Terminally oxidized ω-hydroxy fatty acids (ω-OHFAs) and α,ω-dicarboxylic acids (α,ω-DCAs) are multifunctional compounds useful for the production of polymers and musk fragrances. Polymers derived from saturated and unsaturated ω-OHFAs are regarded as bio-based plastics with high water resistance, durability and chemical versatility (Liu *et al*., 2011). ω-Oxyfunctionalized products derived from medium-chain length fatty acids such as dodecanoic acid (C12-FA) can be used to synthesize different bioplastics. 12-Hydroxydodecanoic acid (ω-OHC12) serves as a building block for the production of poly(12-hydroxydodecanoate). Poly(12-hydroxydodecanoates) can be copolymerized, for instance, with 12-hydroxystearate to yield an environmental benign elastomer (Ebata *et al*., 2008). In this context, cyclododecanone is another industrial relevant key material used for the production of polyamides musk fragrances, crop protection products and pharmaceuticals (Teles *et al*., 2009; Ebel *et al*., 2013). Chemically, ω-OHFAs can be obtained by cross-metathesis of unsaturated fatty acid esters, followed by the hydroformylation and hydrogenation of the carbonyl group (Warwel *et al*., 1992; Metzger and Bornscheuer, 2006) or by the reduction of α,ω-DCAs (Yokota and Watanabe, 1992). Dicarboxylic acids can be prepared by the catalytic ring opening of lactones and cyclic ketones (Cotarca and Nardelli, 1999; Stephan and Mohar, 2006). However, the synthesis of ω-OHFAs or α,ω-DCAs via the terminal oxygenation of saturated or unsaturated fatty acids is not practised in chemocatalysis, as selectivity and controlled reactivity in C-H oxygenation reactions are difficult to achieve. Moreover, chemical methods proven to be successful in terms of productivity and regiospecificity are not sustainable as they rely on harsh reaction conditions and high energy-demanding processes (Labinger and Bercaw, 2002).

Biosynthetic ω-OHFAs and α,ω-DCAs are currently produced via yeast-based technologies (Lu *et al*., 2010). Most existing platforms consist of engineered *Candida* and *Yarrowia* strains that produce selective cytochrome P450 monooxygenases (CYPs), namely from the CYP52 family. CYP52 enzymes introduce one oxygen atom into the unreactive terminal position of alkanes and fatty acids (Craft *et al*., 2003). Although much progress has been done, this production platform has not been exploited to a larger extent yet (Lu *et al*., 2010). Factors affecting the industrialization of these processes are low space-time yields, biocatalyst recycling and the requirement of new biotechnological production facilities.

With the goal of establishing an alternative bacterial-based process to produce valuable ω-oxyfunctionalized fatty acids, we selected one candidate from the CYP153A enzyme subfamily, whose members have been described as alkane hydroxylases with high terminal regioselectivities (Maier *et al*., 2001; van Beilen *et al*., 2006; Bordeaux *et al*., 2012). Similar to CYP52 enzymes, CYP153A monooxygenases exhibit alkane or fatty acid ω-hydroxylase activity. We have been able to demonstrate that CYP153A from *Polaromonas* sp. (CYP153A_*P*. sp._) is a predominantly alkane ω-hydroxylase, whereas CYP153A16 from *Mycobacterium marinum* and CYP153A from *Marinobacter aquaeolei* (CYP153A*_M. aq._*) are alkane, primary alcohol and fatty acid ω-hydroxylases with preference towards the latter compounds (Scheps *et al*., 2011). Recently, CYP153A*_M. aq._* was found to be a broad substrate-ranged fatty acid ω-hydroxylase, offering high flexibility for the syntheses of ω-OHFAs of different size and saturation level (Honda Malca *et al*., 2012). This biocatalyst oxidized saturated C_12:0_-C_14:0_ and monounsaturated C_16:1_-C_18:1_ substrates with high conversions (63–93%) and ω-regioselectivities (more than 95%). Hot spots involved in substrate selectivity/specificity were identified, with variant G307A in CYP153A*_M. aq._* displaying increased catalytic efficiency towards medium-chain fatty acids in comparison with the wild-type enzyme (Honda Malca *et al*., 2012).

Native CYP153A enzymes depend on a ferredoxin (Fdx) reductase and a Fdx to obtain reducing equivalents from NAD(P)H. In whole-cell biotransformations with recombinant proteins, the catalytic efficiency of the monooxygenase might be impaired by low expression levels of the separate redox proteins or their inability to interact in equimolar ratios with the CYP enzyme (Witholt *et al*., 1990). The redox components might also be expressed as apoproteins (not functional) because they fail to incorporate or retain the corresponding prosthetic group (FAD, FMN or [2Fe-2S]). In this sense, linking the different components in a functional fusion protein represents an adequate solution to improve the efficiency of electron transfer for increased substrate oxidation (Hlavica, 2009). One important point is the higher protein level that could be achieved due to a better coordination of the transcription and translation levels along with the fact that these proteins are encoded by a single gene instead of three different ones. Additionally, fine tuning of only one existing complex is a simpler task, especially when an enzyme cascade is applied (Munro *et al*., 2007). The Pfor reductase component contains a FMN-binding domain, a NAD(P)H-binding domain and a [2Fe-2S] Fdx domain (Liu *et al*., 2006). Misawa and coworkers fused CYP153A from *Acinetobacter* sp. OC4 or CYP153A13a from *Alcanivorax borkumensis* SK2 to the Pfor of P450RhF from *Rhodococcus* sp. NCIMB 9784 to improve the activity of the enzymatic system and carried out whole-cell biotransformations in *Escherichia coli* (Nodate *et al*., 2006; Fujita *et al*., 2009). Later, the catalytic parameters and *in vivo* activities of the CYP153A13a-Pfor construct towards different substrates were further investigated (Bordeaux *et al*., 2011). This construct showed the highest turnover frequency (57 min^−1^), coupling (54%) and *in vivo* product yield (61 mg 1-octanol g_cdw_^–1^) with *n*-octane. Several other examples of highly active self-sufficient P450-Pfor fusion constructs have been reported. These include P450cam (CYP101) and P450PikC (CYP107L1) as heme domains (Nodate *et al*., 2006; Li *et al*., 2007; Robin *et al*., 2009).

Another interesting reductase component for the creation of fusion CYP chimeras is that of the fatty acid hydroxylase P450 BM3 from *Bacillus megaterium*. P450 BM3 is a self-sufficient enzyme with the highest catalytic activity known for bacterial P450 monooxygenases. The reductase consists of a FAD-containing region that is related to a Fdx reductase and an FMN-binding section similar to a flavodoxin (Joyce *et al*., 2012). A more stable variant could obtained from Eiben and coworkers when they replaced the P450 reductase (CPR) of CYP102A1 with CYP102A3 (Eiben *et al*., 2007). Fused to the CPR of P450 BM3 the human, membrane-bound P450 2E1 could be solubilized, expressed in a bacterial system and biochemically characterized (Gilardi *et al*., 2002). Another concept in this context includes the establishment of a triple fusion of the two natural redox proteins and the heme domain to one functional polypeptide chain. In this light, P450cam can be fused to a functional PdR-Pdx-P450cam (CYP101). CYP175A1 is reported to show as CYP175A1-Fdx-FNR a higher catalytic activity in comparison to the non-fused variant (Sibbesen *et al*., 1996; Mandai *et al*., 2011).

Strain-related factors directly influencing the efficiency of a biocatalytic process include tolerance towards the substrate and product(s) obtained, stable recombinant protein expression, high NAD(P)H regeneration rate, low by-product formation and the possibility of reuse for multiple reaction cycles (Meyer *et al*., 2006). *Escherichia coli* has been described as a suitable bacterial host for oxygenase-based reactions, including those mediated by CYP153A enzymes (van Beilen *et al*., 2005; Fujita *et al*., 2009; Koch *et al*., 2009). *Escherichia coli* offers the advantages of simple genetic manipulation and its application in high-cell-density processes. Furthermore, higher specific activities and product yields on carbon source have been obtained with *E. coli* compared with other production hosts like *Pseudomonas* in reactions catalysed by heterologous monooxygenases (Grant *et al*., 2011; Kuhn *et al*., 2012). Other limiting factors in whole-cell-mediated biotransformations are biocatalyst performance (in terms of kinetics, coupling and stability) and substrate uptake. Former studies with medium- and long-chain aliphatic compounds evidenced that the rate of substrate transport across the cell membrane into the cell is one of the major bottlenecks in the reaction rate (Schneider *et al*., 1998; Grant *et al*., 2012). It has been shown that the coexpression of the alkL gene of *Pseudomonas putida* GPo1 can significantly increase the efficiency of uptake of aliphatic compounds by *E. coli.* This improved substrate uptake has been attributed to the outer membrane transporter AlkL, which was coexpressed with the AlkB monooxygenase in *E. coli* to improve productivities in fatty acid biotransformation experiments (Julsing *et al*., 2012b).

In this study, we describe the creation of fusion proteins comprised by CYP153A*_M. aq._* with the reductase components of P450 BM3 from *B. megaterium* (CPR) or CYP116B3 from *Rhodococcus ruber* (Pfor). We show for the first time the application of an engineered CYP153A fusion construct in the synthesis of ω-OHC12 by *E. coli* strains. Further strain improvement was performed with the implementation of the additional AlkL transporter. Bioprocess development led us to obtain the desired product ω-OHC12 from C12-fatty acid methyl ester (FAME) with high regioselectivity in the gram per litre scale.

## Results and discussion

### Characterization of CYP153A*_M. aq._* with different redox systems

C12-FA was selected as model substrate for this study, given the industrial relevance of the corresponding ω-oxyfunctionalized product. We demonstrated before that C12-FA is also one of the preferred substrates of CYP153A_*M. aq*._ (63% conversion, 95% ω-regioselectivity) (Honda Malca *et al*., 2012), making it suitable for this *in vivo* approach.

CYP153A*_M. aq._* and each of its putative natural redox partners – the FAD-containing oxidoreductase (FdR) and [2Fe-2S] Fdx – were cloned with a His-tag for expression in *E. coli* BL21(DE3). CYP153A*_M. aq._* was expressed in *E. coli* in functional form in this study. Sodium dodecyl sulfate polyacrylamide gel electrophoresis and spectrophotometrical analyses demonstrated that the Fdx was expressed functional, but that the FAD oxidoreductase was produced as an apoflavoprotein. In addition, the whole operon encoding the three separate protein components (Fdx → CYP153A*_M. aq._* → FdR) was expressed in *E. coli*, and cell lysates were used for *in vitro* reactions with *n*-octane. Low substrate conversion levels (less than 2%) were detected in comparison with previously published *in vitro* results with CamA and CamB [15% substrate conversion (Honda Malca *et al*., 2012)]. Reaction products might have been formed as a result of the interaction of the CYP153A enzyme and redox proteins constitutively expressed in *E. coli* (Scheps *et al*., 2011). The natural redox partners of CYP153A*_M. aq._* could therefore not be used for further investigations in whole *E. coli* cells. To overcome the problem of inefficient electron transfer, we decided to establish a functional self-sufficient CYP153A complex.

**Figure 1:**
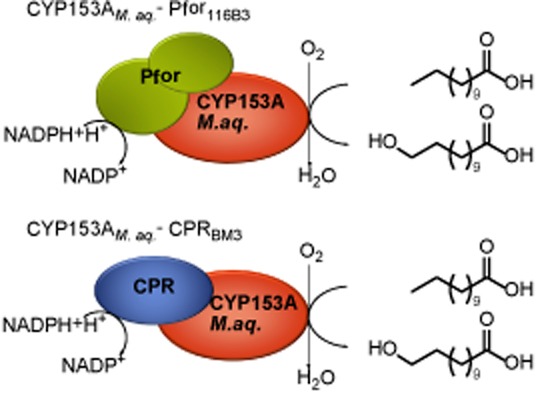
Biotransformations of dodecanoic acid to 12-hydroxy dodecanoic acid with catalytic self-sufficient (A) CYP153A*_M. aq._-*Pfor_116B3_ and (B) CYP153A*_M. aq._-*CPR_BM3_.

We designed fusion constructs containing two different types of C-terminally linked reductase domains (Fig. 1). The first electron transfer systems are Pfor1 and Pfor2 (Table 1), where the reductase domain (Pfor) is provided by CYP116B3, belonging to the family of phthalate monooxygenases and dioxygenases (Liu *et al*., 2006). Because of the fact that the natural redox partners of CYP153A*_M. aq._* harbour the same cofactors (FAD and [2Fe-2S]) as Pfor, we considered this reductase domain an appropriate candidate. In this study, two variants containing either the native or codon-optimized gene coding for Pfor were created. The latter was used as it was already available in our gene library and known to result in high protein yields in *E. coli*. The second electron transfer system consists of the NADPH-dependent cytochrome P450 reductase (CPR) domain of CYP102A1 (P450 BM3). To increase protein flexibility between the CYP153A*_M. aq._* domain and CPR, variants CPR1 and CPR2 with different linker sequences were designed (Table 1). The linker in CPR1 corresponds to the natural peptide linker found between the heme and reductase domains in P450 BM3. CPR2 contained the same linker, although preceded by a 3xGly-Gly-Ser (GGS) region that is known to be a suitable peptide for the connection of different protein domains (Robinson and Sauer, 1998; McNew *et al*., 1999). The two variants were created on the basis that the insertion or deletion of residues in the linker region can affect the activity of the enzyme (Robin *et al*., 2009). A previous study has shown that electron transfer from a eukaryotic CPR (from *Candida apicola*) to a bacterial P450 domain (CYP109B1) is feasible (Girhard *et al*., 2012). In addition, the combination of a bacterial CPR with a eukaryotic P450 domain to express these proteins in soluble form has been already reported (Gilardi *et al*., 2002). To our knowledge, this is the first time that the heme domain of a bacterial class I P450 has been fused to a CPR and demonstrated to be catalytically active.

**Table 1:** Overview of the different used CYP153A self-sufficient fusion constructs

	Subunits of self-sufficient CYP153A*_M. aq._* fusion constructs		
	
Construct name	Heme domain	Linker region	Reductase domain
Pfor1	CYP153A*_M. aq._*	native	natPfor_116B3_^a^
Pfor2	CYP153A*_M. aq._*	imp^b^	impPfor_116B3_^b^
CPR1	CYP153A*_M. aq._*	native	CPR_BM3_
CPR2	CYP153A*_M. aq._*	native + 3xGGS	CPR_BM3_
CPR2_mut_	CYP153A*_M. aq._*_(G307A)_	native + 3xGGS	CPR_BM3_

Strain name	*E. coli* strain	Plasmid	Construct/gene	Experiment
JM109-CPR2	*E. coli* JM109	pJOE	CPR2	Shaking flasks with C12-FA as substrate
JM109-CPR2_mut_	*E. coli* JM109	pJOE	CPR2_mut_	Shaking flasks with C12-FA as substrate
HMS174-CPR2_mut_	*E. coli* HMS174	pET28a(+)	CPR2_mut_	Bioreactor with C12-FA as substrate
HMS174-CPR2_mut_ and AlkL	*E. coli* HMS174	pColaDuet-1	CPR2_mut_ + alkL	Bioreactor with C12-FAME as substrate

CYP153A*_M. aq._* from *Marinobacter aquaeolei* VT8, Pfor_116B3_ from *Rhodococcus ruber* DSM 44319 and CPR_BM3_ from *Bacillus megaterium*.

a nat, native DNA sequence.

b imp, improved codon-optimized DNA sequence.

The N-terminal CYP153A genes were cloned without a purification tag to avoid any negative effect on their activity. All four fusion protein variants were expressed in soluble forms, and their concentrations were determined by the conventional carbon monoxide (CO)-differential spectral assay. The expression level of soluble Pfor2 with the *E. coli* codon-optimized reductase domain was 1.3-fold higher than that of Pfor1; however, a high proportion of the protein was detected in the insoluble fraction. Protein aggregation is prone to trigger stress cell responses (Jurgen *et al*., 2000; Gasser *et al*., 2008), which could have a negative impact on the efficiency of the whole-cell-mediated biocatalytic process. Pfor1 was then used for further comparison experiments with CPR1 and CPR2. We could not detect any difference either in the expression level or in the activity of CPR1 and CPR2 towards the model substrate C12-FA (data not shown). CPR2_mut_ was further utilized because of the reported higher *in vitro* fatty acid ω-hydroxylation activity caused by a single-point mutation (G307A) in CYP153A*_M. aq._* (Honda Malca *et al*., 2012). CPR2 and CPR2_mut_ were here compared under *in vivo* conditions. CPR2_mut_ was later used in bioreactor experiments.

The coupling efficiencies [e.g. NAD(P)H consumption resulting in substrate oxidation] of Pfor1, CPR2 and CPR_mut_ were investigated using *n*-octane, octanoic acid and C12-FA as substrates. CPR2 exhibited 1.6-fold higher coupling efficiency than Pfor1 (Table 2) in the presence of *n*-octane and C12-FA. Although CPR2 showed only 22% coupling efficiency with octanoic acid, this substrate was not converted by the Pfor1 construct. The absence of conversion reflects an inefficient electron transfer, which becomes more evident in the case of non-preferred substrates such as octanoic acid. When assayed with substrate C12-FA, Pfor1 and CPR2 showed, respectively, twofold and 3.3-fold higher coupling efficiencies than that of the artificial system of three separate protein components comprised by CYP153A*_M. aq._*, CamA and CamB in a ratio of 1:5:10 (Table 2). This is not surprising as a better interaction is expected to occur between two fused subunits than among separate proteins because of a close juxtaposition (Munro *et al*., 2007). The electron coupling efficiency of CPR2 is comparable with that reported for CYP153A13a fused to Pfor from *Rhodococcus* sp. NCIMB 9784 in *n*-octane oxidation reactions (Bordeaux *et al*., 2011). Our construct Pfor1, however, resulted in a significantly lower coupling efficiency when using the alkane substrate. Limited interaction between the two subunits cannot be excluded. It was shown before that in contrast to CYP153A13a, CYP153AaciA does not interact well with the redox system Pfor (Fujita *et al*., 2009), which suggests that two highly similar CYP enzymes are not necessarily expected to work with the same type of reductase. Interestingly, our engineered CPR2_mut_ variant (CYP containing mutation G307A) showed a better coupling efficiency than that of CPR2 (wild-type CYP). In addition to the coupling efficiency studies, the hydroxylation activities of Pfor1 and CPR2 were investigated towards C12-FA in shaking flask experiments. Conversion levels of 1 g l^−1^ C12-FA achieved by CPR2 were fourfold higher (427 mg l^−1^ ω-OHC12) compared with the Pfor1-containing strains (105 mg l^−1^ ω-OHC12). Considering these results, CPR2 or CPR2_mut_ were further used in our experiments.

**Table 2:** Coupling efficiencies^a^ (%) of three different CYP153A self-sufficient fusion constructs and the artificial redox system CamA and CamB

	CamA + CamB	Pfor1	CPR2	CPR2_mut_
	
Substrate	Coupling (%)^a^			
*n*-Octane	16	32	52	56
Octanoic acid	n.d.	−	22	n.d.
Dodecanoic acid	18	41	67	73

a The coupling efficiency is defined as the ratio between the product formation rate and the NADPH oxidation rate.

n.d., not determined; –, not detected.

Increasing the coupling efficiencies of the CPR fusion constructs is pivotal for the overall efficiency of whole-cell biotransformations. Coupling efficiencies of less than 75% of the self-sufficient fusion constructs offer room for improvements. To further optimize the coupling efficiency, different strategies could be applied: (i) diversification of the linker length between heme domain and reductase, which can be added in form of additional amino acids to the natural linker sequence. Flitsch and coworkers reported that a variation in the linker length can influence the activity and coupling efficiency of a chimeric fusion protein (Robin *et al*., 2009; 2011). More opportunities of protein movement can increase the chance to accomplish the required conformation. (2) Mutation studies, especially in the active centre helped to stabilize the substrate and to increase the binding affinity. The replacement with bulkier amino acid residues can further lead to the displacement of water molecules and optimization of P450 coupling (Xu *et al*., 2005; Kille *et al*., 2011; Honda Malca *et al*., 2012). At the moment, an exact prediction of the influence of structural aspects on the coupling process in our constructs is not possible. A deeper understanding of the artificial CYP153A-CPR fusion construct might be achieved by analysing the available crystal structure of the FAD/NADPH-binding domain of P450 BM3 (Joyce *et al*., 2012) and that of CYP153A*_M. aq._*, whose three-dimensional structure has not been published yet. Such analysis would allow us to identify interacting specific residues in the CPR and CYP153A domains.

### Shake flask bioconversion of C12-FA by resting *E. coli* cells

Whole-cell processes offer possibilities for biocatalysis that cannot always be met by the use of isolated enzymes. In our case, the enzymatic activity is linked to the capability of an effective and continuous regeneration of cofactors. Furthermore, whole cells confer high oxygenase stability by providing a safe cell compartment with optimal surrounding conditions (Urlacher and Girhard, 2012). Cells also possess the ability to scavenge reactive oxygen species originating from uncoupling reactions, which could damage structural and functional proteins, and thus inactivate the used biocatalyst (Woodley, 2006). In this regard, the use of whole cells is considerably more economic (van Beilen *et al*., 2003).

To keep our experiments simple and reproducible, we selected a resting cell approach being aware of the fact that some aliphatic hydrocarbons, e.g. octanoic acid, cause irreversible damage and cell death to resting cells (Favre-Bulle *et al*., 1991). Medium- and long-chained fatty acids are not toxic for the cells (Marounek *et al*., 2003). In contrast to growing cells, metabolically active resting cells have lower carbon and energy demands, thus the cofactor formed during central carbon catabolism can be exploited more efficiently for biocatalysis than for cell growth (Julsing *et al*., 2012a). In all our experiments, we worked with non-metabolically engineered as well as non-solvent adapted *E. coli* strains.

Bacterial systems prefer mostly specific compounds as energy and carbon source, and upon depletion, they start to utilize alternative ones. In the presence of glucose, *E. coli* is able to repress the expression of other carbon catabolic pathway enzymes like those related to β-oxidation. The biotransformation of shorter fatty acids like octanoic acid can be challenging because of the prevention to utilize glucose in *E. coli*, whereas medium- and long-chain fatty acids cause no effect on the cell metabolism (Marounek *et al*., 2003).

**Figure 2:**
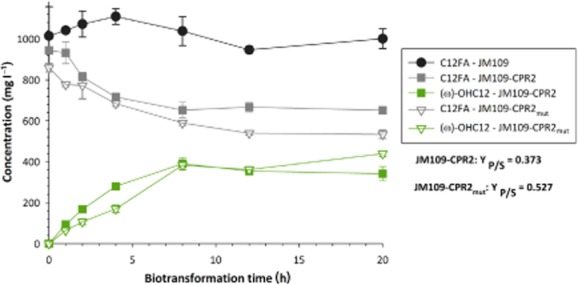
Whole-cell biotransformations of 1 g l^−1^ dodecanoic acid with resting *E. coli* JM109 cells containing empty pJOE, pJOE-CPR2 or pJOE-CPR2_mut_. Cell suspensions were 50 g_cww_ l^−1^. Cells were fed with additional C-source at 0, 4, 8 and 12 h. Product yield coefficients (Y_P__/__S_) correspond to grams of total products per gram substrate.

In this section, the CPR2 and CPR2_mut_ constructs were subcloned into a vector of the L-rhamnose-inducible pJOE series, shown to be effective in the production of heterologous proteins in *E. coli* JM109 (Stumpp *et al*., 2000; Jeske and Altenbuchner, 2009)*.* The CYP expression levels in JM109-CPR2 and JM109-CPR2_mut_ were similar, with CYP concentrations around 30 mg g_cdw_^–1^ (Table S1). Product yields by resting *E. coli* cells containing CPR2 or CPR2_mut_ were compared. *Escherichia coli* cells harbouring the pJOE vector without gene insert (negative control) allowed the measurement of substrate depletion in the absence of the biocatalyst (Fig. 2). Approximately 1 g l^−1^ C12-FA was added to resting cells at the start of the biotransformation along with the glucose/glycerol mix every 4 h in order to diminish substrate consumption for biomass and energy production. During production, these genetically modified strains were subjected to increased oxidative stress due to exposure to C12-FA, which might affect the energy state of the cells. Therefore, through carbon source feeding in time intervals, the resting cells stay metabolically active and maintain the energy charge of 0.5–0.8 (Chapman *et al*., 1971). Strain JM109 containing the empty pJOE vector consumed less than 10% of the fatty acid substrate when the cells were fed with glycerol and glucose. Strains JM109-CPR and JM109-CPR_mut_ produced 11-hydroxydodecanoic acid [(ω-1)-OHC12], ω-OHC12 as well as α,ω-DC12 in varying quantities (Fig. 2). Maximum ω-OHC12 concentrations were quantified after 20 h of biotansformation time, with strains JM109-CPR2 and JM109-CPR2_mut_ producing 342 and 439 mg l^−1^ ω-OHFA respectively. The total product yield after 20 h resulting from the JM109-CPR2_mut_ strain was 41% higher than that obtained from the JM109-CPR2 strain (Fig. 2). Concerning product distribution, both strains yielded similar amounts of ω-OHC12 (97%), (ω-1)-OHC12 (1%) and α,ω-DC12 (2%) (Table S1).

In order to identify potential factors related to the decrease of product formation rate after 8 h of biotransformation time, hydrogen peroxide and acetate were measured. In the JM109-CPR2_mut_ cultures, a concentration of 274 μM of hydrogen peroxide could be detected after 20 h (Fig. S1 or Table S1). Hydrogen peroxide originates from uncoupling reactions due to P450 inefficiency and oxidative stress (Cabiscol *et al*., 2000). *Escherichia coli* can tolerate small hydrogen peroxide concentrations (50 μM) and controls any resulting damages via a repair machinery (Demple and Halbrook, 1983). The high hydrogen peroxide titres could be a reason for the loss of biocatalyst (10–20%) during the reaction procedure. It was reported before that P450s can lose the essential heme group when they are exposed to similar hydrogen peroxide concentrations (Karpetz *et al*., 1988). Additionally, the strain produced 5.0 g l^−1^ acetate in 20 h, which might influence protein stability in the production host (Son *et al*., 2011). It is known that acetate concentrations higher than 2.4 g l^−1^ can destabilize inner cellular proteins and therefore lead to a decrease of biomass (Siriphongphaew *et al*., 2012). It seems to be likely that the high hydrogen peroxide concentrations as well as the amounts of acetate are responsible for the decreased product formation rate after 8 h. This can be caused by substrate or product inhibition, which has been reported in other monooxygenase-based reactions (Lin *et al*., 2001). In parallel with the reported results, we also performed similar experiments with *E. coli* BL21(DE3) in shaking flasks. We observed that this strain produced significantly more α,ω-DC12 than JM109 (10% *vs.* 2%), presumably because of a higher alcohol dehydrogenase/oxidase activity (data not shown). This indicates that the overoxidation from ω-OHFA to α,ω-DCA is not only influenced by the heterologous expressed biocatalyst, but rather by the natural enzymatic machinery in the cell.

### Biotransformations in small-scale fermenters

**Figure 3:**
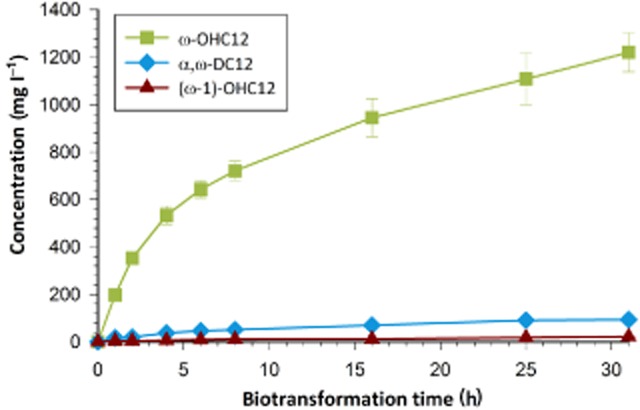
Whole-cell biotransformations of dodecanoic acid with resting cells in a small-scale fermenter. Recombinant strains contained pET-28a(+)-CPR2_mut_.

Because we determined that *E. coli* displayed a higher efficiency during the hydroxylation of fatty acids, conversions were scaled up using 1 l of stirred-tank fed-batch bioreactors. *Escherichia coli* strain HMS174 (DE3) was selected as host as it is a well-known industrial production host. C12-FA (10 g l^−1^) was added at the start of the biotransformation using HMS174-CPR2_mut_ resting cells and 2 g l^−1^ of glucose/glycerol mix after 0, 4, 8 and 12 h. Adequate energy supply was confirmed by online pO_2_ measurements or by analysing random samples via high-performance liquid chromatography (HPLC) afterwards. Thus, we were able to ensure that enough energy source was made available via the feeding strategy. We also suppressed pH regulation that causes a strong foam formation because of alkaline hydrolysis after base addition to the biotransformation mix. We are aware of the fact that rates of fatty acid hydroxylation at a subterminal position by a P450 system can be pH dependent (Su *et al*., 2012), but decided not to adjust the pH to avoid foam formation. The biotransformation resulted in the production of ω-OHC12, with an overall yield of 1.2 g l^−1^ total out of 10 g l^−1^ C12-FA after 30 h. Ninety-two per cent of the total hydroxylated products could be assigned to ω-OHC12. Only 7% of α,ω-DCA and 1% of other hydroxylated products could be detected (Fig. 3, Table S2). However, as high concentrations of hydrogen peroxide and acetate could be determined during shaking flask experiments, it seems to be likely that these factors influence the productivity.

The reduced water solubility of C12-FA is a limiting factor in the biocatalytic process. In addition, toxic substances or inhibitory products that promote unfavourable equilibria impact biotransformations negatively and are a major problem during process development (Lye and Woodley, 1999). A two-liquid-phase concept applied in stirred-tank reactors allows an increased mass transfer by ensuring maximal substrate availability (Cornelissen *et al*., 2011). Formed products like the ω-OHFAs or ω-oxo acids are more hydrophilic as well as reactive than the fatty acids substrates and thus toxic to bacteria. Therefore, their continuous extraction into the hydrophobic layer of a biphasic system can be beneficial (Favre-Bulle *et al*., 1993). FAMEs produced by transesterifications are sometimes more suitable for industrial processes than free fatty acids (FFAs) because of their more advantageous physicochemical properties like occurrence in liquid form and lesser polarity (Leung *et al*., 2010). This gives the possibility of continuous substrate transfer from the organic phase into the aqueous reaction medium, where the reaction itself takes place, coupled with a product removal by extraction that enables the accumulation of toxic oxyfunctionalized products (Marques *et al*., 2009).

We further developed an *E. coli* strain able to express both CPR2_mut_ and AlkL for increased product yields from C12FAME in a biphasic system configuration. Biotransformations were performed in the same reactor setup described before using HMS174-CPR2_mut_ and AlkL, which led to the production of 4.0 g l^−1^ of ω-OHC12 with C12-FAME as substrate applied in a two-phase system (5:1 aqueous/organic phase). The product yields were threefold higher compared with the system lacking AlkL exposed to C12-FA as substrate, with an initial production rate of 21.8 mg/g_cdw_ per hour. Compared with a recent study on the synthesis of ω-hydroxy tetradecanoic acid from methyl tetradecanoate by a CYP52-containing *Candida tropicalis* strain (Lu *et al*., 2010), our value of 4 g l^−1^ after 28 h is lower by a factor of approximately 7.5. However, the applied yeast strain was extensively engineered to minimize substrate or product depletion as well as product overoxidation.

**Figure 4:**
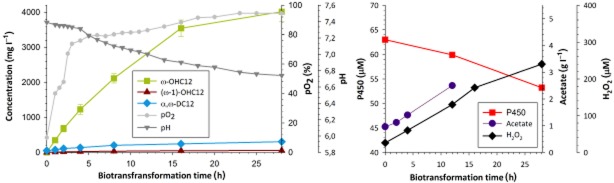
Whole-cell biotransformations of dodecanoic acid methyl ester with resting cells in a small-scale fermenter (left hand). Acetate and hydrogen peroxide formation and P450 concentration over time (right hand). Recombinant strains contained pET-28a(+)-CPR2_mut_. P450, P450 concentration in 1 g_cdw_.

**Figure 5:**
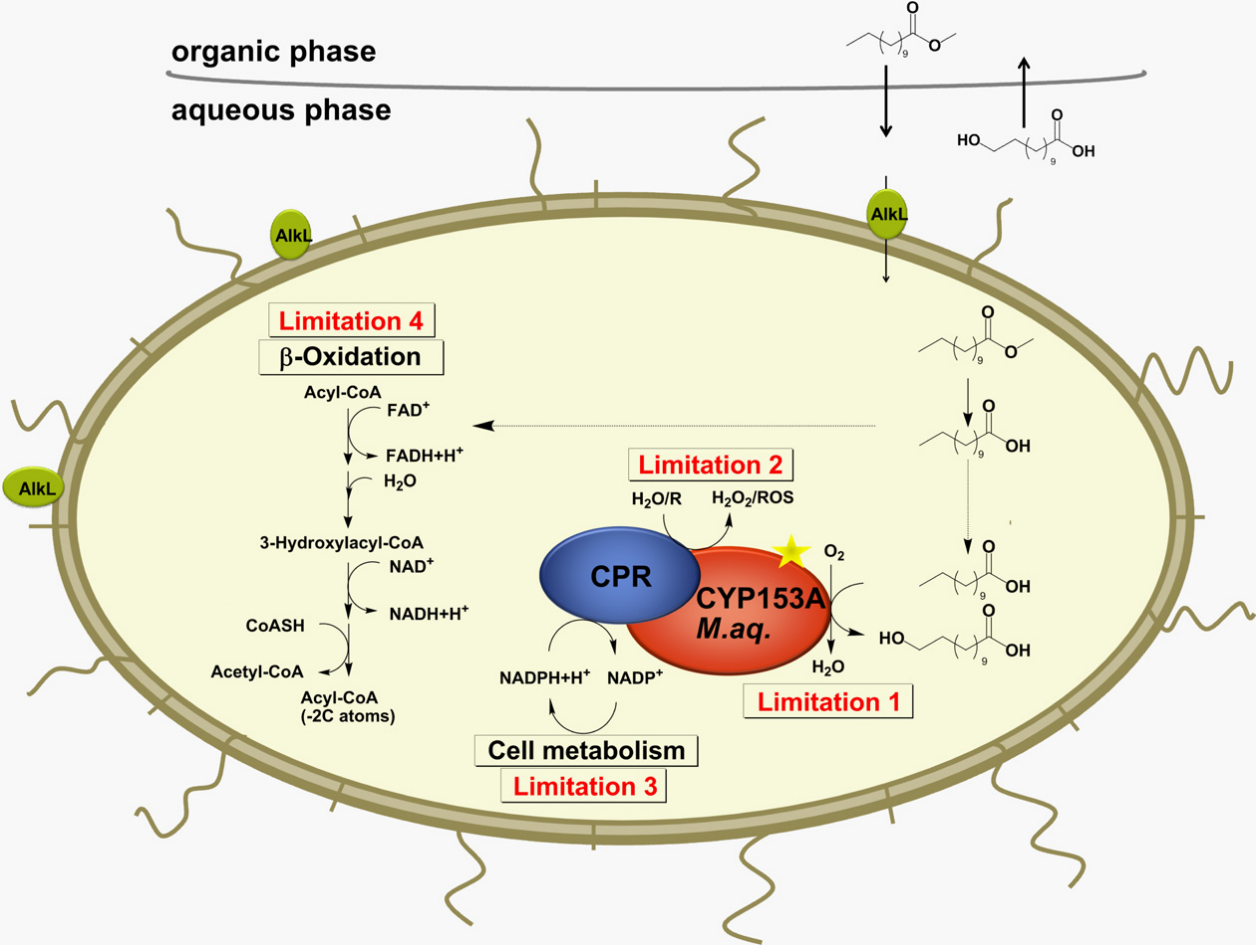
Bottlenecks in ω-hydroxy fatty acid production by recombinant *E. coli in a* two-phase system. (1) CYP153A improvements via engineering approaches to increase the activity and stability of the biocatalyst. (2) Optimization of the coupling efficiency of the applied self-sufficient fusion construct to prevent production of reactive oxygen species (ROS). (3a) Ensurance of sufficient supply of cofactor with an additional NAD(P)H regeneration system or increase of the flux of NAD(P)H-producing reactions. (3b) Restriction in the production of harmful acetate formation by the selection of a suitable C-source feeding strategy or by strain engineering approaches. (3c) Elimination of oxidoreductases responsible for the overoxidation of the formed ω-hydroxylated products to, e.g. α,ω-diacids. (4) Elimination of metabolic pathways to avoid substrate and product depletion (this figure was adapted from Grant *et al*., 2012).

The ω-regioselectivity (including ω-OHFA and α,ω-DCA) of our system was higher than 98%. More than 91% of the formed hydroxylated products consisted of the ω-OHFA (Table S3). Although the product formation rate decreased after 4 h of reaction time (Fig. 3), a plateau was never reached as observed in previous shake flask experiments. The two-phase system configuration with the liquid FAME allows the permanent extraction of the intermediates and the target product, thus minimizing product inhibition and product toxicity. However, once more, hydrogen peroxide and acetate were accumulated in levels close or above the limit for causing a detrimental effect on the cells or on the protein biocatalyst. Hydrogen peroxide was detected in a concentration of up to 240 μM after 28 h, which might have prevented the system to obtain higher product titres (Fig. 4). Acetate levels reached more than 2.2 g l^−1^ after 12 h. The optimization of coupling efficiency and the coexpression of an enzymatic system to degrade reactive oxygen species like hydrogen peroxide to protect the biocatalyst and the metabolic machinery of the cell are essential (Huang *et al*., 2007; Fig. 5). Strategies to improve the production of ω-OHC12 include CYP153A engineering approaches, optimization of the coupling efficiency of the self-sufficient fusion construct, sufficient cofactor and C-source supply as well as elimination hampering oxidoreductases and metabolic pathways (Fig. 5).

### Conclusion and outlook

To conclude, we reported the construction of a catalytically active self-sufficient fusion protein (CYP153A*_M. aq._*-CPR_BM3_) for efficient electron coupling and optimal expression in *E. coli.* The selective ω-hydroxylation of C12-FA as substrate by a non-metabolically engineered as well as non-solvent adapted *E. coli* whole-cell system was shown*.* We could demonstrate a closed mass balance with respect to substrate consumption and product formation. For the highest reported yield of 4 g l^−1^ of ω-OHC12, we used instead of C12-FA its corresponding methyl ester C12-FAME as substrate in combination with the coexpressed outer membrane transport system AlkL.

Until now, the productivity of bacterial fatty acid Ω-hydroxylases has not reached that of yeast CYP52 enzymes. Strain engineering to minimize product overoxidation or substrate consumption when using other commercially relevant fatty acids (e.g., oleic acid) is still necessary. Several other approaches may lead to obtain a flexible bacterial production host in the future (Fig. 5).

## Experimental procedures

### Chemicals, enzymes, vectors and strains

All chemicals, solvents and buffer components were obtained from Sigma-Aldrich (Schnelldorf, Germany). *Pfu* DNA polymerase, endonucleases, *T4* DNA ligase and isopropyl β-D-thiogalactopyranoside (IPTG) were purchased from Fermentas (St Leon-Rot, Germany). Plasmid pET-28a-(+) was purchased from Novagen (Madison, WI, USA). *Escherichia coli* strain DH5α was purchased from Invitrogen (Darmstadt, Germany). *Escherichia coli* strain HMS174(DE3) was kindly provided by the Gerald Striedner lab (BOKU, Vienna, Austria). Plasmid pJOE4782.1 was kindly provided by Josef Altenbuchner (Stumpp *et al*., 2000). Cultivation of *E. coli* was generally performed aerobically in orbital shakers (Multitron; Infors HT, Bottmingen, Switzerland) at 180 r.p.m. and 37°C. Antibiotic concentrations were 30–40 μg ml^−1^ of kanamycin for all cultures. Terrific broth medium supplemented with 6 g l^−1^ glycerol (99%) as carbon source was used for cell growth.

### Cloning of the natural redox partners of CYP153A*_M. aq._*

The monooxygenase CYP153A*_M. aq._* (Maqu_0600) and its putative redox partners, comprised by a FdR (Maqu_0601) and a Fdx (Maqu_0599), were amplified from genomic DNA of *M. aquaeolei* VT8 DSM 11845. Genomic DNA from *M. aquaeolei* VT8 DSM 11845 was purchased from DSMZ (German Collection of Microorganisms and Cell Cultures, Braunschweig, Germany). The CYP enzyme was amplified as previously described (Honda Malca *et al*., 2012). The Fdx was amplified by PCR with oligonucleotides Maq_Fdx_For and Maq_Fdx_Rev (encoding a C-terminal His_6_-tag sequence; Table S4) for its insertion into the NdeI and BamHI cloning sites of pET-28a(+). The FdR gene was amplified with primers Maq_FdR_For and Maq_FdR_Rev (encoding a C-terminal His_6_-tag sequence; Table S4) using a standard PCR protocol for its insertion into the NdeI and SalI cloning sites of the expression vector pET-28a(+). The operon (Maqu_0599 → Maqu_0600 → Maqu_0601) was amplified with the forward primer used for the Fdx and the reverse primer used for the FdR. After a standard digestion and ligation procedure, each plasmid construct was used to transform into competent *E. coli* DH5α cells.

### Construction of chimeric self-sufficient fusion proteins

A multistep cloning strategy was followed to obtain four different CYP153A*_M. aq._* fusion proteins. Pfor1 (CYP153A*_M. aq._*-natPfor_116B3_) consisted of CYP153A*_M. aq._* and the native reductase domain (natPfor_116B3_) of CYP116B3 from *R. ruber* DSM 44319. Pfor2 (CYP153A*_M. aq._*-impPfor_116B3_) consisted of CYP153A*_M. aq._* and a codon-optimized version of the reductase domain (impPfor_116B3_) to achieve high expression levels in *E. coli.* CPR1 (CYP153A*_M. aq._*-CPR_BM3_) was built by fusing CYP153A*_M. aq._* to the reductase domain (CPR_BM3_) of CYP102A1 or P450 BM3 from *B. megaterium*. This construct contained the natural linker sequence found between the heme and the reductase domain of P450 BM3. CPR2 (CYP153A*_M. aq._*-3xGGS-CPR_BM3_) consisted of CYP153A*_M. aq._* fused to CPR_BM3_, with an additional 3xGGS tandem region in the linker sequence.

A standard PCR protocol was used for gene amplification. For Pfor1 (CYP153A*_M. aq._*-natPfor_116B3_), the heme domain was amplified with the forward primer Pfor1_Enz_For and the reverse primer Pfor1_Enz_Rev (Table S4). The natPfor_116B3_ domain was PCR-amplified with the forward primer Pfor1_Red_For and the reverse primer Pfor1_Red_Rev (Table S4). Pfor2 (CYP153A*_M. aq._*-impPfor_116B3_) was obtained by PCR amplification of the heme domain with the same forward primer used for variant 1 and the reverse primer Pfor2_Enz_Rev (Table S4). The impPfor_116B3_ domain was amplified with the forward primer Pfor2_Red_For and the reverse primer Pfor2_Red_Rev (Table S4).

For CPR1 (CYP153A*_M. aq._*-CPR_BM3_), the heme domain was created by PCR amplification with the forward primer CPR1_Enz_For and the reverse primer CPR1_Enz_Rev (Table S4). The CPR_BM3_ component was amplified by PCR with the forward primer CPR1_Red_For and the reverse primer CPR1_Red_Rev (Table S4). For CPR2, the heme domain was amplified with the same forward primer used for CPR1 and the reverse primer CPR2_Enz_Rev (Table S4). To insert the additional 3xGGS tandem region, the CPR_BM3_ domain was amplified with the forward primer CPR2_Red_For and the reverse primer used for CPR_BM3_ amplification in CPR1 (Table S4).

In the following step, the matching amplified products were assembled at their described overlapping sections by PCR and ligated into pET-28a(+). The resulting plasmids were used to transform competent *E. coli* DH5α cells via heat shock. The success of cloning was verified by automated DNA-sequencing (GATC-Biotech, Köln, Germany). Plasmid pET-28a(+) harbouring the chimeric self-sufficient CPR2 construct was subjected to site-directed mutagenesis to introduce mutation G307A as described elsewhere (Honda Malca *et al*., 2012). As a result, CPR2_mut_ (CYP153A_*M. aq.*(G307A)_-3xGGS-CPR_BM3_) was obtained.

### Subcloning of fusion genes into an L-rhamnose-inducible expression system

The CPR2 fusion chimeras were subcloned into a vector of the L-rhamnose-inducible pJOE series for their expression in *E. coli* JM109. pET-28a(+) constructs containing CPR2 or CPR2_mut_ were used as templates for PCR using the forward primer CPR1&2_pJOE_For and the reverse primer CPR1&2_pJOE_Rev (Table S4). Vector pJOE-P450 BM3 derived from pJOE4782.1 was previously constructed in our laboratory (Brass *et al*., 2008; Vomund, 2010). The plasmid was cut with restriction enzymes XbaI and BsrGI to excise the P450 BM3 gene insert. The linearized vector was purified and ligated with the digested PCR-amplified fusion genes. Correct constructs were verified by sequencing (GATC-Biotech). Religated pJOE without insert was later used as negative control.

### Construction of a dual expression vector with alkL and CPR2_mut_

In order to construct a plasmid for the coexpression of alkL and CPR2_mut_, a synthetic gene with the sequence of alkL (Geneart, Regensburg, Germany) and restriction sites BglII and XhoI was used. The CPR2_mut_ section was created via PCR amplification with the forward primer AlkL_For and the reverse primer AlkL_Rev (Table S4). Both gene components were cloned into the dual expression system pCOLADuet-1 using either *Bgl*II and *Xho*I or *Nco*I and *Sac*I restriction enzymes. The correct construction of pColaDuet-1::CPR2_mut_ comprising alkL was confirmed by sequencing (GATC-Biotech).

### Protein expression and purification

Cells were grown in shake flasks until an OD_600_ of 1.0 was reached for induction of protein expression (25°C, 140 r.p.m.). After 16–20 h, cells were collected by centrifugation (10 000 r.p.m., 20 min, 4°C) and resuspended in 50 mM of K_i_PO_4_ (potassium phosphate) buffer pH 7.4 containing 0.1 mM of phenylmethylsulfonyl fluoride (PMSF). For the determination of P450 concentration and electron coupling efficiency, the fusion protein constructs were extracted and purified. Cell pellets were resuspended in 100 mM of K_i_PO_4_ buffer pH 7.4 containing 0.1 mM of PMSF and disrupted in 2–3 cycles on a French press (EmulsiFlex-C5; Avestin, Mannheim, Germany) at 4°C. The resulting crude extracts were centrifuged (37 000 r.p.m., 30 min, 4°C), and the supernatants with the soluble proteins were recovered.

Protein purification was carried out by fast protein liquid chromatography (FPLC; GE Healthcare, Freiburg, Germany) using a column with the weak anion exchange resin Toyopearl DEAE 650 M (TOSOH, Stuttgart, Germany) packed to a volume of 30 ml and a maximum flow rate of 10 ml min^−1^. The column was washed (5 ml min^−1^ working flow) using a step gradient protocol with 25 mM of Tris-HCl buffer pH 7.4 containing 0–1 M of NaCl solution. The elution of the CYP153A fusion proteins occurred at 250 mM of NaCl. In addition to the characteristic total protein detection at 280 nm, CYPs were identified by their absorbance at 418 nm (Sligar *et al*., 1979). This procedure was followed by ultrafiltration using Vivaspin filters with the cutoff size of 100 kDa (Vivaspin 100 kDa; Sartorius, Göttingen, Germany) or by FPLC on Sephacryl S-200 HR (GE Healthcare) to achieve a protein purity of more than 95%. Purified protein solutions were stored in aliquots at −20°C.

### Determination of P450

Concentrations of the P450 enzymes were determined by the CO differential spectral assay based on the formation of the characteristic Fe^II^-CO complex at 448 nm. The cells were disrupted by sonication on ice (4 × 2 min, 2 min intervals). Enzymes in cell-free extracts were reduced by the addition of a spatula tip of sodium dithionite, and the CO complex was formed by slow bubbling with CO gas for approximately 30 s. The concentrations were calculated using the absorbance difference at A_450_ and A_490_ (Ultrospec 3100pro spectrophotometer; GE Healthcare), and an extinction coefficient of 91 M^−1^ cm^−1^ (Omura and Sato, 1964a,b).

### Determination of coupling efficiency

Reaction mixtures (0.8 ml) containing the chimeric self-sufficient CYP153A systems were prepared according to a previous report with a minor modification of 0.5 μM of concentration of CYP (Malca *et al*., 2011). C12-FA was added to a final concentration of 300 μM. *n*-Octane was also assayed for comparison purposes with the existing literature. The reaction was performed at 25°C and started by adding 150 μM or 300 μM NADPH. NADPH consumption was monitored at 340 nm (ε_340_ = 6.22 mM^−1^ cm^−1^) over approximately 15 min. After this procedure, the reaction was stopped by adding 37 % HCl and treated for GC/FID analysis (Malca *et al*., 2011).

### Bioconversions of *n*-octane by CYP153A*_M. aq._* and physiological redox partners

Biotransformations of 1 mM of *n*-octane by CYP153A*_M. aq._* and its natural redox partners were performed as described elsewhere (Scheps *et al*., 2011).

### Shake flask bioconversions of C12-FA by resting *E. coli* cells

One colony of fresh retransformed *E. coli* JM109 or *E. coli* BL21(DE3) cells of each recombinant strain was grown overnight in 5 ml of LB medium with the appropriate antibiotic at 37°C and 180 r.p.m. Two millilitres of the preculture were used to inoculate 400 ml of TB_Kan_ medium. When an OD_600_ of 0.6-0.8 was reached, 0.2 % L-rhamnose was added to induce protein expression. Following the addition of 0.5 mM of 5-aminolevulinic acid, cultures were incubated at 25°C and 180 r.p.m. for 20 h. Cells were harvested at 4228 r.p.m. at 4°C for 25 min, washed twice with 100 mM K_i_PO_4_ buffer pH 7.4 and resuspended in the biotransformation medium.

Shake flask assays with C12-FA were conducted in resting *E. coli* JM109 and *E. coli* BL21(DE3) cells harbouring the pJOE-CPR2 or pJOE-CPR2_mut_ vector constructs. *Escherichia coli* JM109 transformed with empty religated pJOE vector (without gene insert) was used as a negative control to measure substrate depletion in the absence of the biocatalyst.

*In vivo* bioconversions with induced resting cells were carried out in 100 ml shake flasks containing 20 ml of fresh cell suspension (50 g_cww_ l^−1^). The biotransformation medium consisted of 100 mM of K_i_PO_4_ buffer pH 7.4 with 1% (*w*/*v*) glycerol, 0.4% (*w*/*v*) d-glucose, 100 μM of FeSO_4_ and 30–40 μg ml^−1^ of kanamycin. Biotransformations started by the addition of 0.4 ml of a substrate stock solution containing 50 g l^−1^ of C12-FA in DMSO. Reactions were run at 30°C and 180 r.p.m. for 20 h. Cells were fed with a glucose/glycerol mix [0.4% (*w*/*v*) glucose and 1% (*w*/*v*) glycerol] at time points 0, 4, 8 and 12 h. Five hundred microlitre samples were collected at different time points (1, 2, 4, 8, 14 and 20 h after substrate addition). Samples were treated for GC/FID analyses as described in the section ‘Analysis of substrates and formed products’.

### Five litre fed-batch cultivation

A 5 l (operating volume) bioreactor (Infors AG, Bottmanning, Switzerland) containing 4 l of TB_Kan_ medium [per litre deionized water: 12.0 g of tryptone, 24.0 g of yeast extract, 4.0 ml (*v/v*) of glycerol, 2.31 g of KH_2_PO_4_ and 12.54 g of K_2_HPO_4_]. Five millilitres of LB medium was inoculated with freshly transformed *E. coli* HMS174 (DE3) with the CPR2_mut_ fusion constructs. This preculture was used to inoculate a 1 l Erlenmeyer flask containing 250 ml of TB_Kan_ medium, and incubated at 37°C and 180 r.p.m. overnight on an orbital shaker until an OD_600_ of 3–5 was reached. Cultivation in the bioreactor was started by inoculation from the second preculture with necessary volume to a start OD_600_ of 0.05–0.1. The pH was maintained at 7.2 throughout the process using 28% (*v/v*) NH_4_OH and 10% (*v/v*) H_3_PO_4_. The temperature was controlled at 37°C during growth and 25°C after induction of the expression with 0.1 mM of IPTG. The dissolved oxygen content of the culture broth was regulated by variation of the airflow and the agitation speed, and set to approximately 25% during the growth and expression processes.

The expression was started upon reaching an OD_600_ of 9–10. Feeding was kept constant using an 80% (*v/v*) glycerol solution and a speed of 30 g h^−1^ (Pflug *et al*., 2007). Additionally, the medium was supplemented with 5 ml of a 1 M MgSO_4_ × 6H_2_O solution, 500 μl of a 1 M 5-aminolevulinic acid solution, 50 g of (NH_4_)_2_PO_4_, 4 ml of a trace element solution (190 mg CaCl_2_ × 2H_2_O, 90 mg ZnSO_4_ × 7H_2_O, 90 mg CoCl_2_ × 6H_2_O, 75 mg CuSO_4_ × 5H_2_O, 50 mg MnSO_4_ × H_2_O, 11.1 mg Na_2_-EDTA × 2H_2_O and 8.35 mg FeCl_3_ × 6H_2_O in 500 ml of ddH_2_O) and 2 ml of thiamine (100 g l^−1^).

### Biotransformations of C12-FA or C12-FA methyl ester by resting *E. coli* cells

Biotransformations in 1 l (operating volume) bioreactors (Infors AG) were carried out with 450 ml of resting cells solution (50 g_cww_ l^−1^) in 200 mM of K_i_PO_4_ buffer pH 7.4. The biotransformation phase was initiated by addition of 90 ml of C12-FAME. Alternatively, 4.5 g of C12-FA were dissolved in DMSO like described before and added to the biotransformation mix for the reactions with the FFA substrate. The pH was adjusted to 7.4 at the beginning and controlled with an autoclavable amperometric probe (Mettler-Toledo GmbH, Schwerzenbach, Switzerland), but not regulated during the reaction. Likewise, the dissolved oxygen (pO_2_) was not adjusted but monitored during the biotransformation process with an autoclavable amperometric probe (Mettler-Toledo GmbH). Stirrer velocity was set up from 200 (C12-FA) to 800 r.p.m. (C12-FAME). Temperature and aeration rate were maintained at 30°C and 1.5 l min^−1^ respectively.

### Analysis of substrates and formed products

Conversions were stopped with 30 μl of 37% HCl, followed by the addition of decanoic acid as internal standard in a final concentration of 1 mM. The reaction mixtures were extracted twice, with twice the volume of diethyl ether. The organic phases were collected, dried with MgSO_4_ (anhydrous) and evaporated. Samples were resuspended in 60 μl of methyl *tert*-butyl ether, followed by the addition of 60 μl of 1% trimethylchlorosilane in *N*,*O*-bis(trimethylsilyl)trifluoroacetamide and incubation at 75°C for 30 min for derivatization.

Samples were analysed on a GC/FID instrument (Shimadzu, Duisburg, Germany) equipped with a DB-5 column (30 m × 0.25 mm × 0.25 μm; Agilent, Waldbronn, Germany) and with hydrogen as carrier gas (flow rate, 0.8 ml min^−1^; linear velocity 30 cm s^−1^). The injector and detector temperatures were set at 250°C and 310°C respectively. The column oven was set at 130°C for 2 min, raised to 250°C at a rate of 10°C min^−1^, held isotherm for 3 min and then raised to 300°C at 40°C min^−1^. A GC/MS QP-2010 instrument (Shimadzu) equipped with a DB-5 MS column (30 m × 0.25 mm × 0.25 μm, Agilent) and helium as carrier gas was used to identify the products in characteristic samples. The injector and detector temperatures were set at 250°C and 285°C respectively. The column oven temperature programme was the same as that of the GC/FID. Mass spectra were collected using electrospray ionization (70 eV). Reaction products were identified by their characteristic mass fragmentation patterns. Substrate and product conversions were quantified from the GC/FID peaks using calibration curves estimated from a series of standard solutions C12-FA, C12-FAME, ω-OHC12 and α,ω-DCA, treated in the same manner as the samples. ω-Regioselectivities were estimated from the total hydroxylated product.

### Determination of glycerol, glucose and acetate by HPLC analysis

Cells from the fermentation fractions were separated from the supernatant by centrifugation at 20.000 × g for 1 min (Centrifuge 5417 C; Eppendorf, Hamburg, Germany). The supernatant was transferred into a new plastic tube, mixed with the internal standard xylitol to a final concentration of 10 mM and filtered (0.45 μm). HPLC analysis was carried out on an Agilent System (1200 series) using an Aminex HPX-87H Ion Exclusion Column (300 × 7.8 mm, Bio-Rad, Hercules, CA, USA) at 60°C, a mobile phase of 5 mM of H_2_SO_4_ and a flow rate of 0.5 ml min^–1^. The analytes were detected and quantified using the corresponding standards on a refractive index detector (Agilent 1200series, G1262A) set at 35°C.

### Determination of hydrogen peroxide formation in cell crude extracts

Quantitation of total (intracellular and extracellular) H_2_O_2_ was performed by the horseradish peroxidase/phenol/4-aminoantipyrine spectrophotometrical assay as described elsewhere (Xu *et al*., 2005). Reaction mixtures were prepared in 50 mM of Tri-HCl buffer pH 7.5 to a final volume of 800 μl and contained 100 μl of cell culture, 10 μl of BugBuster 10× Protein Extraction Reagent (Novagen, Madison, WI, USA), 12.5 mM of phenol, 1.25 mM of 4-aminoantipyrine and 0.1 mg l^−1^ of horseradish peroxidase. The absorbance of each sample was set at zero before adding the peroxidase. Hydrogen peroxide concentrations were calculated from a calibration curve with known concentrations of H_2_O_2_ (2–80 μM) that yielded absorbances in the linear range. In order to exclude any potential interference of BugBuster with the enzymatic assay, the reagent was added to all standard solutions.

## Supporting information

**Table S1.** Maximum ω-OHFA yields, product distributions and other by-products obtained with resting *E. coli* cells harbouring wild-type CYP153A_*M. aq.*_ or variant CYP153A_*M. aq.*_ G307A fused to the reductase domain of P450 BM3.

**Table S2.** Maximum ω-OHFA yields and product distributions obtained with resting *E. coli* cells harbouring variant CYP153A_*M. aq.*_ G307A fused to the reductase domain of P450 BM3 in a small-scale fermenter using C12-FA.

**Table S3.** Maximum ω-OHFA yields, product distributions and other by-products obtained with resting *E. coli* cells harbouring variant CYP153A_*M. aq.*_ G307A fused to the reductase domain of P450 BM3 in a small-scale fermenter using C12-FAME.

**Table S4.** Oligonucleotide primers used for the construction of CYP153A_*M. aq.*_ self-sufficient fusions, natural redox partners and alkL via PCR.

**Fig. S1.** Acetate and hydrogen peroxide (H_2_O_2_) concentrations in whole-cell biotransformations of dodecanoic acid by resting *E. coli* JM109 cells harbouring the pJOE-CPR2_mut_ vector construct.
