# Supplementary material for: Synthesis of ω-hydroxy dodecanoic acid based on an engineered CYP153A fusion construct
Source: Microb Biotechnol. 2013 Aug 14;6(6):694–707. doi: 10.1111/1751-7915.12073 (PMC3815936; doi:10.1111/1751-7915.12073)
Supplement: Supplementary file 1 [file mbt0006-0694-SD1.doc]

Supplementary Information:

Table S1. Maximum ω-OHFA yields, product distributions and other byproducts obtained with resting *E. coli* cells harboring wild type CYP153A*_M. aq._* or variant CYP153A*_M. aq._* G307A fused to the reductase domain of P450 BM3

| Parameter | Units | *E. coli* JM109 | | |
| --- | --- | --- | --- | --- |
|  |  | CPR2 | | CPR2_mut_ |
| Biotransformation time | h | 20 | | |
| Biomass concentration^1^ | g_cdw_/L | 11.3 | 10.9 | |
| Active P450 concentration^1^ | mg/g_cdw_ | 27.6 | 33.5 | |
| Initial substrate concentration | mg/L | 944 | 859 | |
| Substrate conversion^2^ | % | 34.5 | 48.7 | |
| ω-OHC12 concentration | mg/L | 342 | 439 | |
| α,ω-DC12 concentration | mg/L | 7 | 9 | |
| Product distribution |  |  |  | |
| (ω-1)-OHC12 | % | 1.1 | 0.8 | |
| ω-OHC12 | % | 97 | 97.2 | |
| α,ω-DC12 | % | 1.9 | 2 | |
| Byproducts |  |  |  | |
|  | h |  |  | |
| Acetate [g/L] | 2 | n.d. | 1.7 | |
|  | 8 | n.d. | 3.8 | |
|  | 20 | n.d. | 5.0 | |
| Hydrogen peroxide [µM] | 8 | n.d. | 166 | |
|  | 20 | n.d. | 270 | |

^1^at start of biotransformation. ^2^Substrate converted to oxygenated products (not equivalent to substrate consumption). The values were calculated from initial substrate and product concentrations and represent the molar percentage yields. n.d., not determined

Table S2. Maximum ω-OHFA yields and product distributions obtained with resting *E. coli* cells harboring variant CYP153A*_M. aq._* G307A fused to the reductase domain of P450 BM3 in a small scale fermenter using C12-FA.

| Parameter | Units | *E. coli* HMS 174 (DE3) | | |
| --- | --- | --- | --- | --- |
|  |  | CPR2_mut_ | | |
| Biotransformation time | h | 2 | 8 | 30 |
| Active P450 concentration | mg/g_cdw_ | 63 | 59 | 55 |
| Initial substrate concentration | g/L | 10 | 10 | 10 |
| ω-OHC12 concentration | mg/L | 352 | 721 | 1220 |
| α,ω-DC12 concentration | mg/L | 19 | 52 | 93 |
| Product distribution |  |  |  |  |
| (ω-1)-OHC12 | % | 1 | 1 | 1 |
| ω-OHC12 | % | 94 | 93 | 92 |
| α,ω-DC12 | % | 5 | 6 | 7 |

Biomass concentration was at start of biotransformation 15.1 g_cdw_/L

Table S3. Maximum ω-OHFA yields, product distributions and other byproducts obtained with resting *E. coli* cells harbouring variant CYP153A*_M. aq._* G307A fused to the reductase domain of P450 BM3 in a small scale fermenter using C12-FAME

| Parameter | Units | *E. coli* HMS 174 (DE3) | | |
| --- | --- | --- | --- | --- |
|  |  | CPR2_mut_ + AlkL | | |
| Biotransformation time | h | 2 | 8 | 28 |
| Active P450 concentration | mg/g_cdw_ | 64.5 | 60 | 53.2 |
| Initial substrate concentration | g/L | 174 | 174 | 174 |
| ω-OHFA concentration | mg/L | 676 | 2114 | 4010 |
| α,ω-DCA concentration | mg/L | 108 | 200 | 302 |
| Byproducts |  |  |  |  |
| Acetate | g/L | 1.12 | 2 | 4.5^2^ |
| Hydrogen peroxide | µM | 40 | 80 | 240 |

Biomass concentration was at start of biotransformation 18.2 g_cdw_/L; Initial substrate concentration was 174 g/L; ^2^ estimated from figure 4 (after each indicated time period)

| Primer code | Sequence |
| --- | --- |
| Maq_Fdx_For | GGT CAT ATG ATA TCA TTA ATG GGC GGT CAC GAT GGG CC |
| Maq_Fdx_Rev | ATT GGA TCC TCA ATG ATG ATG ATG ATG ATG CAT CTG GAA CTC GGG TAA ATG |
| Maq_FdR_For | GCC CAT ATG GTA AGC AAA CGT AAA GAG |
| Maq_FdR_Rev | GAT GTC GAC TCA ATG ATG ATG ATG ATG ATG ACT CTG GAG CC |
| Pfor1_Enz_For | GGT CAT ATG ATA TCA TTA ATG CCA ACA CTG CCC AGA ACA TTT GAC G |
| Pfor1_Enz_Rev | CTC GCC GAT GGT GAC GGG ATG CTG CCG TTG CAG CAC ACT GTT CGG TGT CAG TTT GAC CAT CAA CCT GG |
| Pfor1_Red_For | GCA GCA TCC CGT CAC CAT CGG CGA GCC CTC CAC CCG GTC GGT GTC ACG CAC CGT CAC CGT CG |
| Pfor1_Red_Rev | CAT AAG CTT TCA GAG TCG GAG GGT CAG TCG GTC G |
| Pfor2_Enz_For = Pfor1_Enz_For | GGT CAT ATG ATA TCA TTA ATG CCA ACA CTG CCC AGA ACA TTT GAC G |
| Pfor2_Enz_Rev | CAA TGG TAA CCG GAT GCT GAC GCT GCA GAA CAC TGT TCG GTG TCA GTT TGA CCA TCA ACC |
| Pfor2_Red_For | CAT CCG GTT ACC ATT GGT GAA CCG AGC ACC CGT AGC GTT AGC CGT ACC GTT ACC GTT GAA C |
| Pfor2_Red_Rev | CAT AAG CTT TTA CAG ACG CAG GGT CAG ACG ATC ACC |
| CPR1_Enz_For | GGT CCA TGG GTA TGC CAA CAC TGC CCA GAA CAT TTG ACG AC |
| CPR1_Enz_Rev | TAG CAG ACT GTT CAG TGC TAG GTG AAG GAA TAC TGT TCG GTG TCA GTT TGA CCA TCA ACC |
| CPR1_Red_For | CAC TGA ACA GTC TGC TAA AAA AGT ACG CAA AAA GGC AGA AAA CGC TCA TAA TAC GCC GCT GC |
| CPR1_Red_Rev | CAT CTC GAG TTA CCC AGC CCA CAC GTC TTT TGC GTA TC |
| CPR2_Enz_For = CPR1_Enz_For | GGT CCA TGG GTA TGC CAA CAC TGC CCA GAA CAT TTG ACG AC |
| CPR2_Enz_Rev | CTG TTC AGT GCT AGG TGA AGG AAT GCT GCC GCC GCT GCC GCC GCT GCC GCC ACT GTT CGG TGT CAG TTT GAC CAT CAA CC |
| CPR2_Red_For | GCG GCA GCA TTC CTT CAC CTA GCA CTG AAC AGT CTG CTA AAA AAG TAC GCA AAA AGG CAG AAA ACG CTC ATA ATA CGC CGC TGC |
| CPR2_Red_Rev = CPR1_Red_Rev | CAT CTC GAG TTA CCC AGC CCA CAC GTC TTT TGC GTA TC |
| CPR1&2_pJOE_For | C TAG TCT AGA ATG CCA ACA CTG CCC AGA ACA TTT |
| CPR1&2_pJOE_Rev | G TAC TGT ACA TTA CCC AGC CCA CAC GTC TTT |
| AlkL_For | GG TCC ATG GGT ATG CCA ACA CTG CCC AGA ACA TTT GAC GAC |
| AlkL_Rev | CAT GAG CTC TTA CCC AGC CCA CAC GTC TTT TGC GTA TCG GCC |

Table S4. Oligonucleotide primers used for the construction of CYP153A_M.aq_. self-sufficient fusions, natural redox partners and alkL *via* PCR.


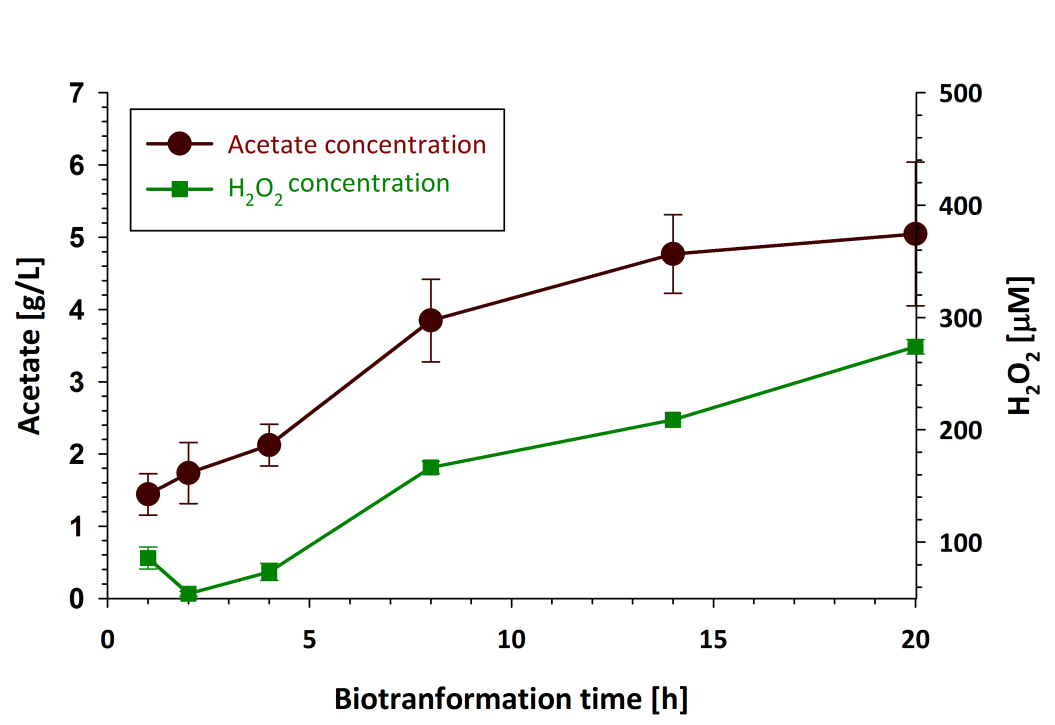


Figure S1. Acetate and hydrogen peroxide (H_2_O_2_) concentrations in whole cell biotransformations of dodecanoic acid by resting *E. coli* JM109 cells harboring the pJOE-CPR2_mut_ vector construct.
